# Poster Session II - A274 PREDICTING RISK FOR VENOUS THROMBOEMBOLISM AFTER HOSPITAL DISCHARGE IN PATIENTS WITH INFLAMMATORY BOWEL DISEASE

**DOI:** 10.1093/jcag/gwaf042.274

**Published:** 2026-02-13

**Authors:** G Tennakoon, J McCurdy, A Seeraj, R Ghasemi, H Chaudhary, S Zhang

**Affiliations:** Temerty School of Medicine, University of Toronto Temerty Faculty of Medicine, Toronto, ON, Canada; The Ottawa Hospital Foundation, Ottawa, ON, Canada; The Ottawa Hospital Foundation, Ottawa, ON, Canada; University of Ottawa Faculty of Medicine, Ottawa, ON, Canada; University of Ottawa Faculty of Medicine, Ottawa, ON, Canada; McGill University Faculty of Medicine and Health Sciences, Montreal, QC, Canada

## Abstract

**Background:**

Patients with inflammatory bowel disease (IBD) face an elevated risk of venous thromboembolism (VTE) during hospitalization. Emerging evidence suggests this risk may persist post-discharge, yet no validated tools exist to identify high-risk patients who may benefit from extended thromboprophylaxis.

**Aims:**

To identify risk factors associated with risk of VTE among patients with IBD patients within 3 months following hospital discharge.

**Methods:**

We performed at retrospective, matched case-control study at The Ottawa Hospital (2009-2024). Adults (>17 years) with pre-existing IBD who developed a VTE were identified by our institutional databases using validated ICD-10 codes, confirmed by chart review. Patients hospitalized ≥48 hours who developed a VTE within 3 months after hospital discharge (cases) were matched to 3 patients without post-dicharge VTE (controls) by discharge date. Candidate predictors were selected a priori based on literature review. Univariable analysis identified variables with p < 0.05. Next, multivariable conditional logistic regression with stratification by matched set was performed.

**Results:**

We identified 468 eligible hospital discharges, 117 eligible post-discharges were identified occurring within 3 months prior to a VTE event, matched to 351 controls (Table 1). Extended thromboprophylaxis was only given to 8.1% of discharges. Multivariable analysis identified five independent predictors (Figure 1): prior VTE (OR 5.60; 95% CI 2.95-10.62), central venous catheter during admission (OR 5.09; 2.14-12.07), cancer (past/current) (OR 3.41; 1.45-8.02), hemoglobin <100 g/L (OR 2.43; 1.32-4.46), and age >45 years (OR 2.32; 1.23-4.39) (all p < 0.01).

**Conclusions:**

In this single-center retrospective study, we identified multiple clinical variables that are associated with VTE after hospital discharge in patients with IBD. This work will help to create a future multicenter clinical prediction tool to identify which patients with IBD have the highest risk of developing VTE after hospital discharge.

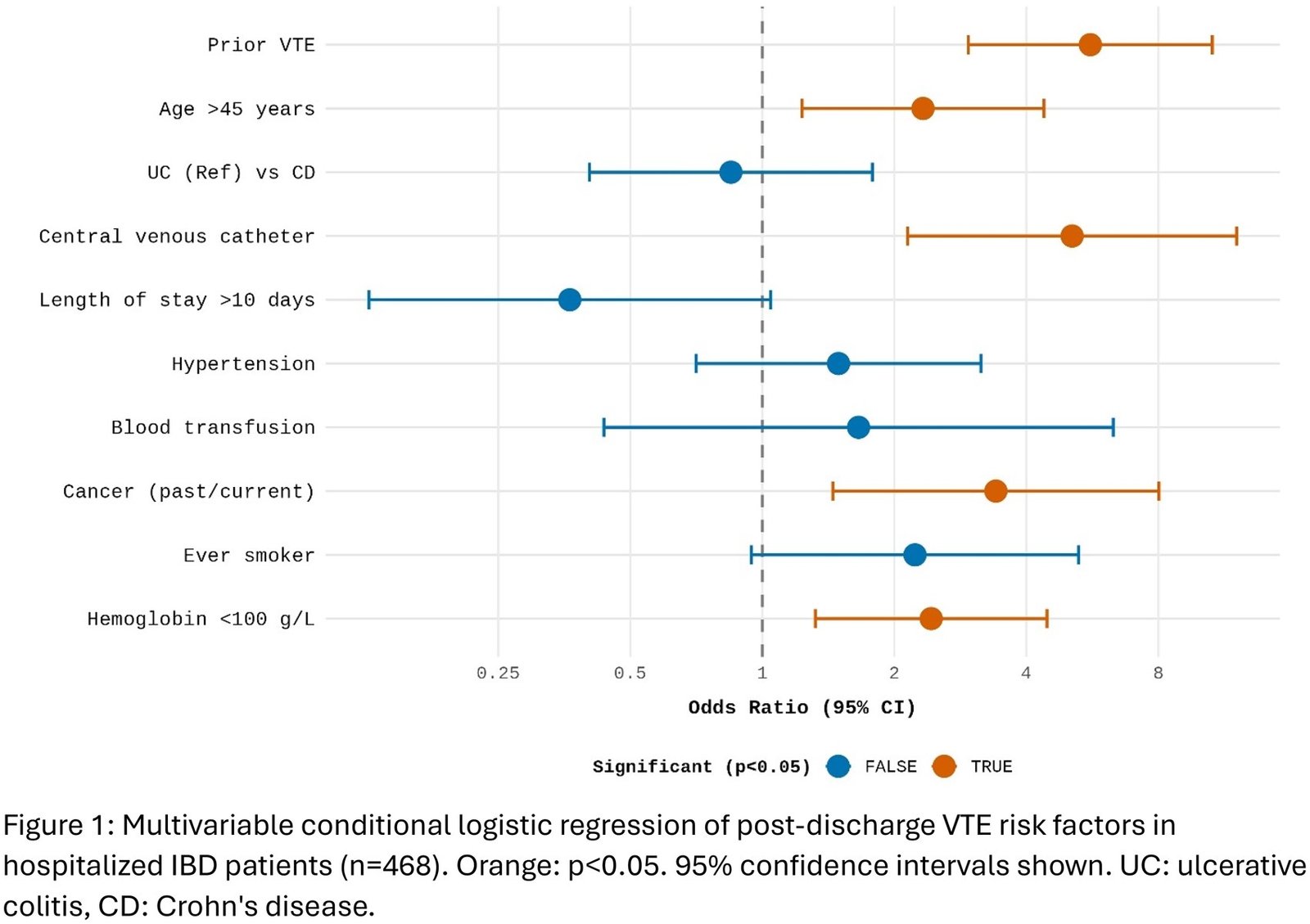

**Funding Agencies:**

Ottawa Hospital Research Institute

**A274 Table 1:** Descriptive statistics and univariable analysis of IBD patients with and without post-discharge VTE.

Characteristic	Controls (n = 351)	Cases (n = 117)	P value
Female, n (%)	179 (51.1)	57 (48.7)	0.737
Age, mean years (SD)	47.8 (18.6)	54.0 (14.6)	<0.001
Prior VTE, n (%)	27 (7.7)	61 (52.1)	<0.001
Hypertension, n (%)	73 (20.9)	43 (36.8)	0.001
Diabetes, n (%)	43 (12.2)	23 (19.7)	0.071
Cancer (past/current), n (%)	59 (16.7)	38 (32.5)	0.002
Smoker (past/current), n (%)	84 (24.1)	42 (35.9)	0.021
Crohn’s disease, n (%)	222 (63.3)	85 (72.6)	0.09
Ulcerative colitis, n (%)	129 (36.7)	32 (27.4)	0.09
Length of stay, mean days (SD)	9.3 (12.4)	12.2 (18.5)	0.121
Central venous catheter, n (%)	34 (9.6)	39 (33.3)	<0.001
Blood transfusion, n (%)	40 (11.3)	28 (23.9)	0.002
Lowest hemoglobin, mean g/L (SD)	110.6 (26.4)	93.8 (22.1)	<0.001

